# Understanding Health Deterioration and the Dynamic Relationship between Physical Ability and Cognition among a Cohort of Danish Nonagenarians

**DOI:** 10.1155/2020/4704305

**Published:** 2020-06-03

**Authors:** Cosmo Strozza, Virginia Zarulli, Viviana Egidi

**Affiliations:** ^1^Interdisciplinary Centre on Population Dynamics, University of Southern Denmark, 5000 Odense, Denmark; ^2^Department of Statistical Sciences, Sapienza University of Rome, 00185 Rome, Italy

## Abstract

This study aims to determine how demographics, socioeconomic characteristics, and lifestyle affect physical and cognitive health transitions among nonagenarians, whether these transitions follow the same patterns, and how each dimension affects the transitions of the other. We applied a multistate model for panel data to 2262 individuals over a 2-year follow-up period from the 1905 Danish Cohort survey. Within two years from baseline, the transition probability from good to bad physical health—ability to stand up from a chair—was higher than dying directly (29% vs. 25%), while this was not observed for cognition (24% vs. 27%) evaluated with Mini-Mental State Examination—a score lower than 24 indicates poor cognitive health. Probability of dying either from bad physical or cognitive health condition was 50%. Health transitions were associated with sex, education, living alone, body mass index, and physical activity. Physical and cognitive indicators were associated with deterioration of cognitive and physical status, respectively, and with survivorship from a bad health condition. We conclude that physical and cognitive health deteriorated differently among nonagenarians, even if they were related to similar sociodemographic and lifestyle characteristics and resulted dynamically related with each other.

## 1. Introduction

The proportion of the oldest-old has increased during the last decades as a consequence of the decline in old-age mortality [1–3]. The share of nonagenarians in Denmark increased from around 0.08% in 1950 to 0.82% in 2020 and is expected to further grow during the next years, reaching 2.03% in 2050 [4]. This phenomenon is taking place in most developed countries, fueling a growing interest on the health conditions of oldest-old [2]. Health transitions at older ages are of particular interest as deterioration of both physical and cognitive health conditions is very likely [5, 6].

Physical and cognitive health decline have been investigated, in order to understand whether they can be partially explained by other health characteristics [7]. A systematic review of the relationship between physical functioning and cognition was published by Clouston et al. in 2013 [8], which found that physical functioning at baseline was associated with longitudinal changes in cognition but the opposite relationship was inconsistent. Physical mobility and functioning dynamically interact between healthy and unhealthy states [5, 9–11]. Cognitive health declines with age more linearly, even though this decline can cover a more complex pattern [6, 12, 13]. It is therefore crucial to investigate further how the physical and cognitive deteriorations evolve and whether they follow different patterns. The relationship between the two dimensions of the health status has been widely investigated. However, the literature lacks studies on the oldest-old and this is why in this study we focus on individuals aged 90+.

With this analysis, we aim at investigating (1) how demographic and socioeconomic characteristics and lifestyle habits affect transitions in physical and cognitive health; (2) whether these transitions follow the same patterns; and (3) how does each dimension (physical or cognitive) affect the transitions of the other dimension. To our knowledge, this is the first study that analyzes the relationship between physical and cognitive decline and the determinants of transitions in these two health dimensions (physical and cognitive) among nonagenarians.

## 2. Methods

### 2.1. Study Population and Measures

The study population comes from the 1905 Danish Cohort survey, which contains many individual level information on the members of the cohort born in Denmark in 1905 interviewed and tested for physical and mental health in their home by a survey agency. It is a longitudinal multiassessment survey conducted from 1998 to 2005 with four waves realized every 2‐3 years. Detailed information about the study design are available in Nybo and colleagues [14]. In this work we use the first two waves of the Danish 1905 Cohort Survey, collected in 1998 and 2000, when the oldest-old were, respectively, 93 and 95 years old. The initial population, corresponding to the study population, was composed of 2626 individuals. They represent 62.8% of the potential participants: individuals born in 1905 and living in Denmark. At the second data collection in 2000, 874 were found to be dead (38.6%) reducing to 1388 individuals the number of potential participants to the second wave of the study. The final population interviewed in 2000 was composed of 1086 individuals (78.2% of the potential participants).

The cognitive function was measured with the Mini-Mental State Examination (MMSE): the higher the score (0–30), the better the cognitive status [15]. We grouped it into three standard categories, in order to distinguish people with severe (0–17), mild (18–23), and no cognitive impairment (24–30) based on the most frequently used categorization in literature [16, 17]. The physical function was assessed by the Chair-Stand test: the elderly who can stand up from a chair have better functional status than who need to use hands or cannot do it. This test was found to be a good predictor of disability and mortality among the elderly other than a proper instrument to measure lower body strength [17–20].

We dichotomized both health indicators, in order to create two categories: healthy and unhealthy oldest-old. Regarding cognitive health, individuals were considered cognitively impaired when reporting a MMSE score from 0 to 23 and not cognitively impaired when the score was between 24 and 30. Regarding physical ability, individuals who were not able to stand up from the chair, even with aids, were considered in bad physical health while individuals able to stand up from the chair, with and without use of aids, were considered in good physical health.

Demographic and socioeconomic characteristics (sex, education, and living conditions), critical events (loss of a close relative or friend), health characteristics and behaviors (self-rated health and depression, smoking habits, body mass index (BMI), physical activity, and use of medications) were considered as confounders and controlled in the analysis. Education was used to measure the socioeconomic status of the participants. It was grouped into three categories: (1) elementary school; (2) vocational education; and (3) higher education. Living condition was divided into people living (1) alone and (2) with someone. The loss of a close person, self-rated health, and depression were used to assess the general health perception of the participant and the feelings related to it. The loss of a close person was categorized into two classes: (1) lost someone (spouse, sons, and close friends) and (2) no people lost due to death within the last five years. Self-rated health was assessed with the first question of Short-Form 12 (SF12) questionnaire [21]: ”How do you consider your health in general?”. It was grouped in three categories: (1) very poor or poor; (2) acceptable; and (3) good or excellent. Depression was assessed using an adaptation of the depression section of the Cambridge mental disorders of the elderly examination [22]. It uses a scale from 17 to 52 and it was grouped into three equal-size categories: (1) 17–22; (2) 23–28; and (3) 29–52. Among the health behaviors, smoking habit was categorized into (1) never smoked; (2) past smoker; and (3) current smoker. BMI was calculated on the basis of the reported height and weight at the interview and categorized into three groups: (1) < 22; (2) 22–28; and (3) > 28. Physical activity was assessed by asking if they were performing light (light gardening, short walks, or bicycle rides) or heavy (heavy gardening, long walks or bicycle rides, sports, gymnastics, or dancing) exercises at the time of the interview. It was grouped into three categories: (1) never or not able; (2) light physical activity; and (3) heavy physical activity. The number of medications (daily intake) was coded according to the Anatomical Therapeutic Chemical classification system and it was grouped into three equal-size categories: (1) 0‐1; (2) 2‐3; and (3) 4+.

The main reference for variable selection and classification is Appendix S1 of the article by Thinggaard et al. (2016) that uses the same study population [17]. The proportion of dropouts is 13.4%. We performed a sensitivity analysis in order to check whether dropout was associated with bad health and we did not find any significant association with both health indicators.

### 2.2. Statistical Analysis

We applied a multistate model for panel data—with Markov chain assumption—[23, 24] to assess the association between the many potential drivers measured on the Danish nonagenarians and the probability of transitioning from one health state to another (defined as transition probability). The possible transitions are from good to bad health status, from good health to death, and from bad health to death.

The multistate model we used is based on a stochastic multistate process (*X*(*t*), *t* ∈ *T*) with a finite state-space *S*  =  {1,  ...,  *N*}, where *T*  =  [0,  *τ*],  *τ* < *∞* represents the time (discrete, for panel data). It is fully characterized through transition probabilities between states *h* and *j*:

(1) $$\begin{array}{c} p_{hj}\left(s,t\right)=P\left(X\left(t\right)=j|X\left(s\right)=h\right). \end{array}$$

for *h*, *j* ∈ *S*,  *t*, *s* ∈ *T* or through transition intensities:

(2) $$\begin{array}{c} \alpha_{hj}\left(t\right)=\underset{\Delta t⟶0}{\text{lim}}\frac{p_{hj}\left(t,t+\Delta t\right)}{\Delta t}, \end{array}$$

representing the instantaneous hazard of progression to state *j* conditionally on occupying state *h* at the previous time. According to the Markov assumption, the probability of the next transition depends only on the state occupied at the time *t*.

The effect of the explanatory variables *z*_*it*_ on the transition intensity for individual *i* at time *t* is modeled using proportional intensities, replacing *α*_*hj*_ with

(3) $$\begin{array}{c} \alpha_{hj}\left(z_{it}\right)=\alpha_{hj}^{0}\text{exp}\left(\beta_{hj}^{T}z_{it}\right). \end{array}$$

We conducted the analysis separately for physical and cognitive health, in order to be able to include the baseline status of each dimension (cognitive or physical resp.) as potential driver in the model for the transitions related to the other dimension.

States have been defined according to the MMSE, when assessing cognition, and according to the Chair-Stand test, when the focus was on the physical status. Based on both classifications, we divided participants into two groups based on their good or bad health condition.

Transitions between four states (good health, bad health, nonparticipant but alive, and nonparticipant because dead) have been estimated through transition probabilities. We evaluated the effect of the covariates on the transition intensities only for the “worsening” transitions: from good to bad health condition, from good health condition to death, and from bad health condition to death. As expected, only few people experienced “improving” transitions, as this is unlikely at very old ages.

Because of the relatively small number of individuals in analysis, we could only use the dichotomic classification of MMSE and Chair-Stand test, as the sample size was too small to estimate the coefficients with a finer classification of the variables. We could not perform the analysis separately for men and women due to the small number of nonagenarian men in the sample.

We used methods of imputation with survey data [25] to deal with missing at random values. More information about the imputation method is available in Supplementary Text S1.

Statistical analysis was performed using *R* version 3.5.0 [26].

## 3. Results

### 3.1. Descriptive Results

Of the 2262—93 years old—baseline participants of the study, one-fourth were men (25.8%) while the rest of the people were women (74.2%).

Men had, on average, a higher education level than women, especially in terms of vocational education (32.9% of men vs. 14.2% of women). Fewer men were living alone compared to women (50.5% of men vs. 64.4% of women).

More men experienced the loss of a close person (spouse, children, and close friends) due to death during the last five years (71.7% of men vs. 66.9% of women) but they reported lower rates of depression (39.0% of men vs. 32.3% of women were not depressed) without declaring better health conditions than women (12.5 % of men rated their health as good or excellent while 14.2 % of women did it).

In terms of health behaviors, except for the higher share of (past or current) smokers (78.8% of men vs. 32.4% of women), men had higher BMI (73.1% of men vs. 55.3% of women had a BMI higher than 22) and performed more physical activity (43.8% of men vs. 28.9% of women perform some physical activity) than women. More details about baseline characteristics of the population are available in Table 1.

Men scored better in terms of cognitive (48.5% of men vs. 40.6% of women were not cognitively impaired) and physical (52.1% of men vs. 41.5% of women were able to stand up from the chair without any aid) health compared to women as reported in Table 2.

### 3.2. Multi-State Analysis Results

We analyzed physical and cognitive health deterioration in two different models including, respectively, cognitive and physical baseline health status because the main aim of the study is to examine the dynamic relationship between these two health aspects and not because we considered them independent. This implies that part of the individuals in the different states of the two analyzes are the same, resulting in similar transition probabilities and covariates associated with transition intensities.

At baseline, 44.2% of the individuals were in good physical health while 42.7% were in good cognitive health. After two years, 38.6% of the study population died while 13.4% dropped out from the study.

The probability of moving from a good to a bad physical health condition within two years was higher than dying directly (29% vs. 25%). People in bad physical health condition have a 50% probability of dying from a bad physical health status within two years.

**Figure 1:**
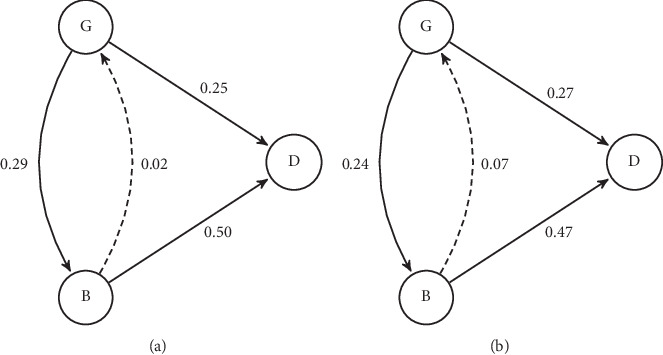
Transition probabilities of the multistate model where states are defined according to (a) physical health and (b) cognitive health. Note. (G) good health status; (B) bad health status; (D) dead.

When considering the cognitive health, the results showed a different pattern. The probability of worsening a good cognitive health condition was lower than experiencing death directly within two years (24% vs. 27%), while individuals in a bad cognitive health have a 47% probability of dying from that condition in the next two years as shown in Figure 1.

The complete transition probabilities are available in Supplementary Tables S1 and S2.

**Figure 2:**
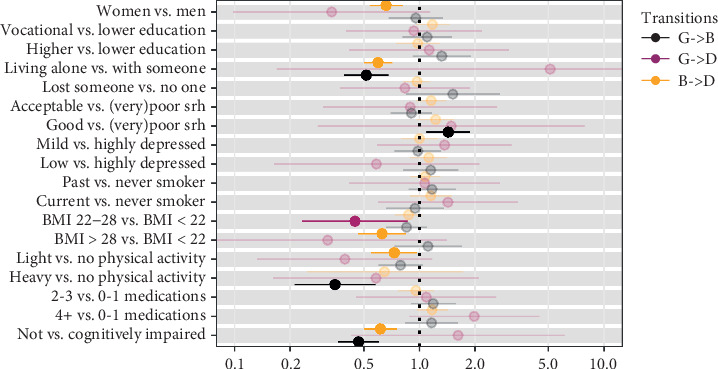
Multivariate predictions (hazard ratios) of transitions in physical health. *Note.* Highlighted hazards ratios are significant; (G) good health status; (B) bad health status; (D) dead.

**Figure 3:**
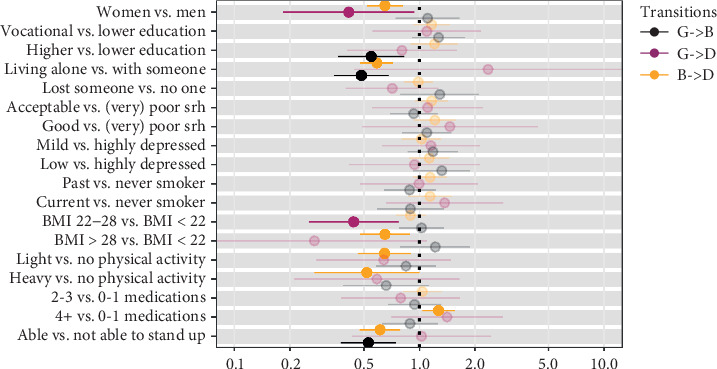
Multivariate predictions (hazard ratios) of transitions in cognitive health. *Note.* Highlighted hazards ratios are significant; (G) good health status; (B) bad health status; (D) dead.

The effect of covariates on the transition intensities is reported in Figures 2 and 3 for the physical states and cognitive states, respectively.

Full details about the two models are available in Supplementary Tables S3 and S4.

#### 3.2.1. Physical Health Transitions

Being women was associated with lower probability of dying for people in bad physical health (female vs. male HR = 0.66) as well as living alone (living alone vs. with someone HR = 0.60). Living alone was also significantly associated with a lower probability of transitioning from a good to a bad physical health (HR = 0.52). Having a BMI higher than 22 statistically decreased the probability of dying, both from a good (BMI 22–28 vs. < 22: HR = 0.45) and a bad (BMI > 28 vs. < 22: HR = 0.63) physical health. Performing physical activity lowered the transition probability from good to bad physical health (heavy vs. no physical activity: HR = 0.35) and from bad physical health to death (light vs. no physical activity: HR = 0.73). Finally, also being cognitively not impaired was statistically associated with a lower probability of worsening the physical health (HR = 0.47) and dying from a bad one (HR = 0.62).

#### 3.2.2. Cognitive Health Transitions

Being a woman was associated with a lower probability of death (from good health: HR = 0.42; from bad health: HR = 0.65). Having higher level of education decreased the probability of deteriorating the cognitive health (HR = 0.55) as well as living alone (HR = 0.49), which was also a protective factor against transitioning from bad cognitive status to death (HR = 0.59). BMI higher than 22 reduced the probability of dying from a good (BMI 22–28 vs. < 22: HR = 0.44) and a bad (BMI > 28 vs. < 22: HR = 0.65) cognitive health. Doing physical activity was significantly related to lower transition rates from bad to death (light vs. no physical activity: HR = 0.65, heavy vs. no physical activity: HR = 0.52). As expected, using more than four medications per day was associated with higher probability of death when already in a bad cognitive health (HR = 1.27). Finally, being able to stand up from the chair without any aid was statistically associated with a lower probability of worsening the cognitive health (HR = 0.53) and dying from a bad one (HR = 0.61).

## 4. Discussion

The increasing proportion of the oldest-old people in the last decades increased the attention of researchers and policy makers on this subgroup of individuals [2, 3]. As physical and cognitive health are two dynamic processes and their deterioration is likely, especially at older ages, in the recent years it became a widely investigated topic [5, 6,9, 10, 12]. Finding the determinants of physical and cognitive health changes and analyzing their longitudinal relationship are considered, nowadays, two of the major public health challenges [27, 28]. However, only few studies analyzed such deteriorations among the oldest-old [8, 29]. Studying the determinants of physical and cognitive health transitions among very old people and analyzing the relationship between these two conditions will help to shed light on which are the most vulnerable groups.

This study uses two waves of the 1905 Danish Cohort survey [14] to study the transitions in physical and cognitive health among individuals aged 93 at the baseline (1998) and 95 at the second wave (2000). Studies on this cohort showed that high level of disability and poor cognitive and physical performance are strong predictors of mortality in the oldest-old [30, 31]. More precisely, Thinggaad et al. (2016) found that being able to stand up from a chair and having a good level of cognition increased the probability of surviving to age of 100 for both women and men of the 1905 Danish Cohort Study [17].

Our results partially confirm the trends shown in the literature for both physical and cognitive health over the years among adults and younger elderly [7, 9, 11, 29, 32]. Even at very old ages, for individuals in good physical health conditions, the probability of dying directly was lower than the probability of first experiencing a health deterioration. This is what we called here a “one-step worsening pattern.” However, this pattern was not observed for cognitive health in which the probability of deteriorating the level of cognition was lower than dying directly from a good cognitive status (24% vs. 27%).

The analysis of potential drivers of the health decline showed similar results for physical and cognitive health, showing that the two dimensions of the health status follow somewhat similar patterns. However, it is important to point out that this might also partly be due to the overlap of individuals in good and bad state for both physical and cognitive health.

Demographic and socioeconomic variables in both cases resulted associated with health transitions. Not surprisingly, women had a lower probability of death [33–35]. However, by analyzing physical and cognitive dimension separately, we were able to uncover interesting dynamics. Being a woman did not affect significantly the transition from good to bad health. However, it was instead associated with a lower probability of dying from both good and bad cognitive status but only lowered the probability of dying from a bad physical health condition. As expected, having a higher level of education decreased the probability of cognitive decline, confirming the results found among younger adults [12, 32, 36, 37]. However, we found that the level of education did not affect the physical status, contrary to what has been found for a similarly aged (8 years younger) cohort of Canadian elderly [11, 29]. Living alone is widely considered a predictor of physical [9, 10, 29] but not for cognitive health transitions. In our study, instead, we found that living alone affected both dimensions of the health status by decreasing the probability of deterioration. Anyway, it was not possible to disentangle the causal direction of the association (whether individuals in better health conditions are able to live alone or whether living alone helps protecting the health condition).

Surprisingly, emotional characteristics did not have significant effect on any of the health transitions analyzed here, despite the fact that other scholars found that self-rated health and depression have an active role in explaining transitions in physical and cognitive health among old individuals [7, 29, 38, 39].

For both health conditions, having a BMI higher than 22 (both categories “22–28” and “>28”) resulted in lower probability of dying both from a good and a bad health status, confirming previous findings on younger adults [40, 41] and in mortality research [42]. Light to moderate exercise was significantly associated with lower probability of dying from both bad physical and cognitive status, while engaging in heavy physical activity was associated with a lower risk of deterioration of the physical health condition and a lower chance of dying when already in bad cognitive status. According to the instrument used by Nybo et al. [14], the level of physical activity is related to the ability of performing Activities of Daily Living (ADL). Other studies reported this association in terms of physical frailty [5, 9, 10] for disability transitions while only little is known about the association between physical exercise and cognitive transitions [43]. As in the case of the living arrangement, it was not possible to distinguish the causal direction of the association between physical activity and physical health.

## 5. Conclusions

Our study sheds light on the dynamic relationship between physical and cognitive conditions among a cohort of nonagenarians, highlighting a “one-step worsening” pattern in physical health, which has not been shown before among nonagenarians. However, we did not observe the same pattern for cognition: individuals in good cognitive status at baseline are slightly more likely to die within two years compared to the first experience deterioration of their cognition. The strengths of this study are the sample size and the extensive information available, which is rare to find given the age (93 years old) of the individuals under analysis. This made it possible to control for many covariates. The weakness of this study is that, even though the data set is longitudinal, it was not possible to clearly identify the causal relationship of some of the associations.

Transitions in both health dimensions were related to similar sociodemographic and behavioral characteristics, with some interesting exceptions, but, surprisingly, not to emotional factors. The two health dimensions resulted associated with each other in terms of transitions: being in a better health condition according to one of the two health measures lowered the probability of worsening the other health status or dying from a bad condition. This confirms what have been discussed by the extensive literature review by Clouston and colleagues [8] about the role of the physical condition at baseline on the transitions in cognitive health and brings new evidence on the role of the cognitive status on the transitions in physical health for which the literature so far has not found consistent evidence.

## Figures and Tables

**Table 1:** Characteristics of the study population in the first wave in 1998 when the individuals were 93 years old.

Characteristics	Sex						*p*
	M		F		T		
	*n*	%	*n*	%	*n*	%	
*Sample*	584	25.8	1678	74.2	2262	100.0	

*Education*							<0.001
Elementary	292	50.0	1254	74.7	1546	68.3
Vocational	192	32.9	238	14.2	430	19.0
Higher	100	17.1	186	11.1	286	12.6

*Living alone*							<0.001
No	289	49.5	598	35.6	887	39.2
Yes	295	50.5	1080	64.4	1375	60.8

*Loss of a close person*							0.033
No	165	28.3	556	33.1	721	31.9
Yes	419	71.7	1122	66.9	1541	68.1

*Self-rated health*							0.013
Very poor or poor	307	52.6	886	52.8	1193	52.7
Acceptable	204	34.9	553	33.0	757	33.5
Good or excellent	73	12.5	239	14.2	312	13.8

*Depression*							0.008
29–52	184	31.5	591	35.2	775	34.3
23–28	172	29.5	545	32.5	717	31.7
17–22	228	39.0	542	32.3	770	34.0

*Smoke*							<0.001
Current smoker	144	24.7	171	10.2	315	13.9
Past smoker	316	54.1	372	22.2	688	30.4
Never smoked	124	21.2	1135	67.6	1259	55.7

*Body Mass Index*							<0.001
< 22	157	26.9	750	44.7	907	40.1
22–28	348	59.6	785	46.8	1133	50.1
> 28	79	13.5	143	8.5	222	9.8

*Physical activity*							<0.001
None/irrelevant	328	56.2	1193	71.1	1521	67.2
Light	177	30.3	390	23.2	567	25.1
Heavy	79	13.5	95	5.7	174	7.7

*Number of medications*							0.057
4+	228	39.0	714	42.6	942	41.6
2‐3	153	26.2	423	25.2	576	25.5
0‐1	203	34.8	541	32.2	744	32.9

Men versus women from Pearson *χ*^2^ test.

**Table 2:** Health conditions of the study population in the first wave in 1998 when the individuals were 93 years old.

Characteristics	Sex						*p*
	M		F		T		
	*n*	%	*n*	%	*n*	%	
*Physical ability: Chair-Stand Test*							<0.001
Not able	70	12.0	293	17.5	363	16.0
With use of hands	210	36.0	689	41.1	899	39.7
Without use of hands	304	52.1	696	41.5	1000	44.2

*Cognitive health: Mini-Mental State Examination*							<0.001
0–17	124	21.2	472	28.1	596	26.3
18–23	177	30.3	524	31.2	701	31.0
24–30	283	48.5	682	40.6	965	42.7

Men versus women from Pearson *χ*^2^ test.

## Data Availability

The data that support the findings of this study are from *The 1905 Danish Cohort Study*, but restrictions apply to the availability of these data, which were used under license for the current study, and so they are not publicly available.

## Supplementary Materials

Supplementary Materials Multistate analysis tables: S1–S4. Supplementary table S1 : transition probabilities of the multistate model where states are defined according to the physical health. Supplementary Table S2 : transition probabilities of the multistate model where states are defined according to the cognitive health. Supplementary Table S3 : multivariate predictions (hazard ratios) of transitions in physical health. Supplementary Table S4 : multivariate predictions (hazard ratios) of transitions in cognitive health.
