# Supplementary material for: Understanding Health Deterioration and the Dynamic Relationship between Physical Ability and Cognition among a Cohort of Danish Nonagenarians
Source: J Aging Res. 2020 Jun 3;2020:4704305. doi: 10.1155/2020/4704305 (PMC7323846; doi:10.1155/2020/4704305)
Supplement: Supplementary Materials — Multistate analysis tables: S1–S4. Supplementary table S1 : transition probabilities of the multistate model where states are defined according to the physical health. Supplementary Table S2 : transition probabilities of the multistate model where states are defined according to the cognitive health. Supplementary Table S3 : multivariate predictions (hazard ratios) of transitions in physical health. Supplementary Table S4 : multivariate predictions (hazard ratios) of transitions in cognitive health. [file 4704305.f1.pdf]

Supplementary materials

Supplementary text S1: missing data imputation

Multiple imputation was necessary to deal with missing at random values (MAR) in order to avoid loss of precision in the analysis. Following the literature on imputation with survey data, we used K-nearest neighbor imputation method (Chen & Shao, 2000). Taking advantage of all the variables available in the dataset except for the one analyzed, we considered five neighbors to calculate the aggregated values to impute.

Multi state analysis tables

Supplementary table S1: Transition probabilities of the multi-state model where states are defined according to the physical health*

From/To	Good	Bad	Dropout	Dead
Good	0.34	0.29	0.13	0.25
Bad	0.02	0.34	0.14	0.50

*Health status according to Chair-Stand Test

Supplementary Table S2: Transition probabilities of the multi-state model where states are defined according to the cognitive health*

From/To	Good	Bad	Dropout	Dead
Good	0.37	0.24	0.12	0.27
Bad	0.07	0.31	0.14	0.47

*Health status according to Mini-Mental State Examination

Supplementary Table S3: Multivariate predictions (hazard ratios) of transitions in physical health

Covariates	From	Good						Bad		
	To	Bad			Dead			Dead		
		HR	IC95%L	IC95%U	Dead	IC95%L	IC95%U	Dead	IC95%L	IC95%U
Sex (Ref. Men)	Women	0.96	0.68	1.34	0.34	0.10	1.15	0.66	0.54	0.82
Education (Ref. Lower)	Vocational	1.10	0.81	1.50	0.93	0.40	2.19	1.18	0.96	1.45
	Higher	1.32	0.92	1.91	1.13	0.42	3.07	0.98	0.75	1.29
Living alone (Ref. No)	Yes	0.52	0.39	0.68	5.11	0.17	154.80	0.60	0.50	0.71
People lost (Ref. No)	Yes	0.91	0.70	1.17	0.83	0.37	1.88	0.97	0.82	1.15
Self-rated health (Ref. Poor)	Acceptable	1.44	1.09	1.89	0.89	0.30	2.65	1.16	0.96	1.40
	Good	1.52	0.85	2.73	1.49	0.28	7.85	1.22	0.97	1.55
Depression (Ref. 29-52)	23-28	0.98	0.73	1.32	1.37	0.59	3.16	1.00	0.79	1.26
	17-22	1.16	0.82	1.63	0.59	0.16	2.11	1.12	0.89	1.42
Smoke (Ref. Never smoked)	Past	1.17	0.87	1.58	1.07	0.42	2.75	1.08	0.89	1.30
	Current	0.95	0.66	1.36	1.43	0.60	3.42	1.15	0.89	1.49
Body Mass Index (Ref. <22)	22-28	0.85	0.66	1.10	0.45	0.23	0.87	0.88	0.74	1.04
	>28	1.11	0.73	1.70	0.32	0.07	1.41	0.63	0.47	0.85
Physical Activity (Ref. None)	Light	0.79	0.60	1.04	0.39	0.13	1.18	0.73	0.55	0.97
	Heavy	0.35	0.21	0.58	0.58	0.16	2.10	0.65	0.25	1.72
Medications (Ref. 4+)	2 3	1.19	0.90	1.58	1.09	0.45	2.61	0.96	0.76	1.20
	0 1	1.17	0.84	1.62	1.99	0.88	4.47	1.18	0.97	1.42
Mmse (Ref. 0-23)	24-30	0.47	0.36	0.61	1.62	0.43	6.13	0.62	0.50	0.76

Supplementary Table S4: Multivariate predictions (hazard ratios) of transitions in cognitive health

Covariates	From	Good						Bad		
	To	Bad			Dead			Dead		
		HR	IC95%L	IC95%U	Dead	IC95%L	IC95%U	Dead	IC95%L	IC95%U
Sex (Ref. Men)	Women	1.11	0.74	1.65	0.42	0.18	0.94	0.65	0.52	0.82
Education (Ref. Lower)	Vocational	1.27	0.92	1.76	1.10	0.56	2.16	1.16	0.93	1.46
	Higher	0.55	0.36	0.83	0.81	0.41	1.60	1.21	0.90	1.62
Living alone (Ref. No)	Yes	0.49	0.35	0.68	2.36	0.45	12.45	0.59	0.48	0.72
People lost (Ref. No)	Yes	0.94	0.69	1.26	0.71	0.40	1.27	0.99	0.83	1.18
Self-rated health (Ref. Poor)	Acceptable	1.10	0.81	1.49	1.11	0.55	2.21	1.17	0.95	1.44
	Good	1.29	0.79	2.10	1.47	0.49	4.42	1.21	0.94	1.57
Depression (Ref. 29-52)	23-28	1.18	0.87	1.62	1.16	0.63	2.13	1.03	0.80	1.32
	17-22	1.32	0.92	1.89	0.94	0.42	2.13	1.13	0.88	1.46
Smoke (Ref. Never smoked)	Past	0.89	0.64	1.23	1.00	0.48	2.09	1.14	0.92	1.41
	Current	0.90	0.59	1.37	1.37	0.66	2.85	1.15	0.87	1.51
Body Mass Index (Ref. <22)	22-28	1.03	0.78	1.36	0.44	0.25	0.77	0.90	0.75	1.08
	>28	1.22	0.79	1.89	0.27	0.07	1.11	0.65	0.48	0.89
Physical Activity (Ref. None)	Light	0.85	0.58	1.23	0.64	0.28	1.48	0.65	0.47	0.90
	Heavy	0.66	0.39	1.13	0.59	0.21	1.65	0.52	0.27	1.00
Medications (Ref. 4+)	2 3	0.94	0.68	1.31	0.79	0.38	1.66	1.04	0.82	1.32
	0 1	0.89	0.63	1.26	1.42	0.71	2.84	1.27	1.04	1.56
Chair-Stand (Ref. Not able)	24-30	0.53	0.38	0.75	1.03	0.43	2.45	0.61	0.48	0.79
